# Hepatitis C Virus—Core Antigen: Implications in Diagnostic, Treatment Monitoring and Clinical Outcomes

**DOI:** 10.3390/v16121863

**Published:** 2024-11-29

**Authors:** Duong Hoang Huy Le, Sitthichai Kanokudom, Ha Minh Nguyen, Ritthideach Yorsaeng, Sittisak Honsawek, Sompong Vongpunsawad, Yong Poovorawan

**Affiliations:** 1 Center of Excellence in Clinical Virology, Department of Pediatrics, Faculty of Medicine, Chulalongkorn University, Bangkok 10330, Thailand; huyldh@pnt.edu.vn (D.H.H.L.); sitthichai.k@chula.ac.th (S.K.); ritthideach.yor@gmail.com (R.Y.); sompong.vo@chula.ac.th (S.V.); 2 Center of Excellence in Osteoarthritis and Musculoskeleton, Faculty of Medicine, Chulalongkorn University, King Chulalongkorn Memorial Hospital, Thai Red Cross Society, Bangkok 10330, Thailand; sittisak.h@chula.ac.th; 3 Medical Biochemistry & Molecular Biology Department, Fundamental Sciences and Basic Medical Sciences, Pham Ngoc Thach University of Medicine, Ho Chi Minh City 700000, Vietnam; nguyenminhha@pnt.edu.vn; 4 Laboratory Department, Nguyen Tri Phuong Hospital, Ho Chi Minh City 700000, Vietnam; 5 The Royal Society of Thailand, Sanam Sueapa, Bangkok 10330, Thailand

**Keywords:** hepatitis C virus, HCV core antigen, diagnostics, HCV treatment, monitoring, resource-limited settings, global elimination strategies

## Abstract

The hepatitis C virus (HCV) infection, a global health concern, can lead to chronic liver disease. The HCV core antigen (HCVcAg), a viral protein essential for replication, offers a cost-effective alternative to HCV RNA testing, particularly in resource-limited settings. This review explores the significance of HCVcAg, a key protein in the hepatitis C virus, examining its structure, function, and role in the viral life cycle. It also evaluates its clinical use in diagnosis and treatment monitoring, comparing its performance to the standard HCV RNA assay using data from PubMed and Google Scholar. HCVcAg assays show high pooled sensitivity (93.5%) and pooled specificity (99.2%) compared to HCV RNA assays, correlating closely (*r* = 0.87) with HCV RNA levels. Hence, HCVcAg testing offers a cost-effective way to diagnose active HCV infections and monitor treatment, especially in resource-limited settings, but its sensitivity can vary and standardization is needed. HCVcAg also predicts liver disease progression and assesses liver damage risk, aiding patient management. It helps to identify patients at risk for fibrosis or carcinoma, making it vital in hepatitis C care. HCVcAg testing can expand access to HCV care, simplify management, and contribute to global elimination strategies, especially in low- and middle-income countries.

## 1. Introduction

### 1.1. A Brief Overview of HCV

The hepatitis C virus (HCV) is a blood-borne pathogen that primarily infects the liver. Transmission occurs through exposure to infected blood, primarily via contaminated needles, unscreened blood transfusions, and high-risk sexual practices. Acute HCV infection often goes unnoticed, due to mild or absent symptoms [1]. However, chronic infection can lead to severe liver complications, including cirrhosis, liver failure, and hepatocellular carcinoma (HCC). As a consequence, HCV infection is a significant global health concern, contributing to an estimated 290,000 deaths annually [2]. Previously considered incurable, advancements in direct-acting antivirals (DAAs) therapies have revolutionized HCV treatment, achieving cure rates exceeding 95% in many cases [3]. Achieving the World Health Organization (WHO) policy for hepatitis C elimination goals by 2030, however, requires a multifaceted public health strategy that includes prevention, screening, diagnostic, and treatment programs tailored to the needs of key affected populations.

### 1.2. Structure of HCV Virion

**Figure 1:**
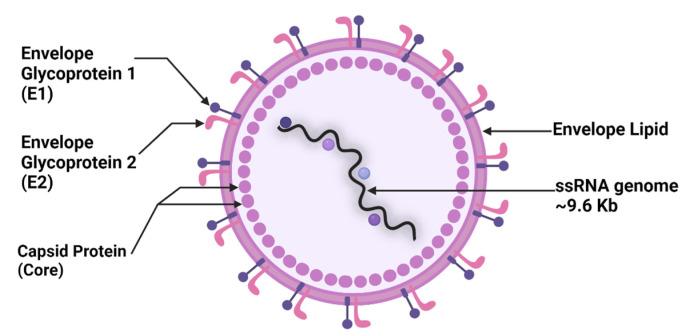
Structure of HCV. E: envelope glycoprotein; ssRNA: single-stranded RNA. Created with https://www.biorender.com/ (accessed on 8 September 2024).

The HCV virion is a small, enveloped virus with a positive-sense, single-stranded RNA (ssRNA) genome. The HCV capsid is entirely formed by the core protein, which adopts an icosahedral shape. The inner surface of the capsid binds the ssRNA genetic material, while the outer surface interacts with the viral membrane or envelope. The envelope consists of a lipid bilayer and two glycoproteins, E1 and E2 (Figure 1). The E1 and E2 proteins are responsible for viral attachment and entry into host cells [4].

### 1.3. HCV Genome

The HCV genome consists of a single-stranded, positive-sense RNA molecule approximately 9600 nucleotides long. This genome encodes a single open reading frame (ORF) of 3010 amino acids, flanked by 5′ and 3′ untranslated regions (UTRs) essential for viral replication and translation. The 5′ NTR contains an internal ribosome entry site (IRES). The translation of the HCV genome begins at the 5′ UTR internal ribosome entry site (IRES), producing a single polyprotein. Cellular proteases cleave this polyprotein to generate structural proteins (core, E1, and E2) that form viral particles. Viral proteases further process the polyprotein to produce non-structural proteins (p7, NS2, NS3, NS4A, NS4B, NS5A, and NS5B) [5].

### 1.4. Variability of HCV

HCV exhibits significant genetic diversity due to the error-prone nature of its RNA-dependent RNA polymerase, which lacks 5′ to 3′ exonuclease activity for proofreading. This results in frequent nucleotide substitutions and contributes to the high degree of genetic variability observed in HCV populations. The mean frequency of nucleotide mutations varies from 1.4 × 10^3^ to 1.9 × 10^3^ substitutions per nucleotide and per year [5,6]. Rapid mutation in HCV creates a diverse population of viruses, known as quasispecies. These quasispecies are classified by differences in specific parts of the virus’s envelope protein and nonstructural 5A protein [7].

HCV genome conservation varies across different regions. Regions encoding essential functions (e.g., translation, replication) or exhibiting structural constraints (e.g., 5′ and 3′ UTRs) show high conservation. The 5′ UTR demonstrates the highest conservation (>90% sequence identity) even among distantly related strains. The capsid-encoding region also exhibits considerable conservation (approximately 80% identity). Conversely, the envelope glycoprotein-encoding region displays significant variability, particularly within the hypervariable regions (HVR1 and HVR2) of E2, where inter-strain sequence identity can be as low as 50%. This variability likely contributes to immune evasion and viral persistence. Structural studies suggest that mechanisms may include point mutations within the epitope, allosteric modulation of the epitope by distal mutations, and glycan shifting on envelope glycoproteins. Consequently, the polypeptides encoded by these hypervariable regions are highly tolerant of amino acid substitutions [8].

**Figure 2:**
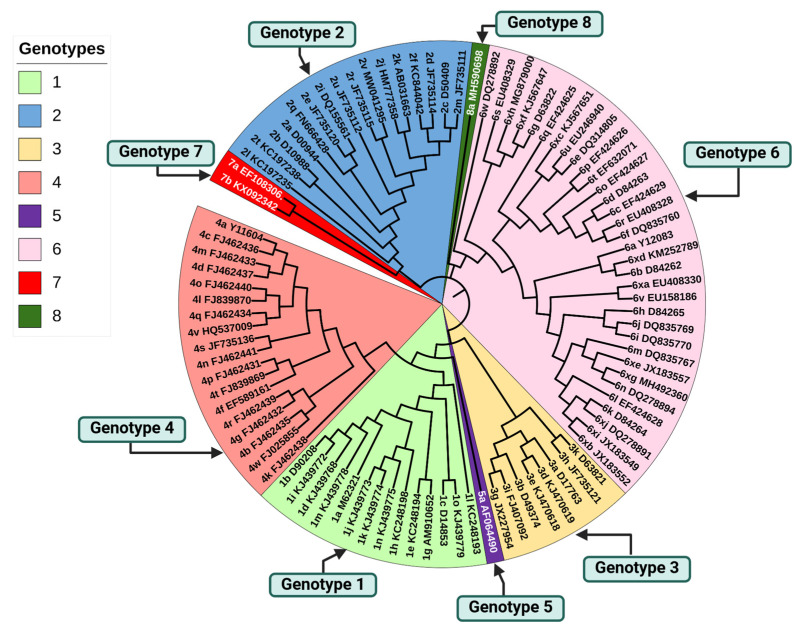
Phylogenetic tree of the eight HCV genotypes based on genomes from the NCBI Genbank. Sequences were aligned using MEGA v.11 software, and the tree was constructed with the maximum likelihood method based on the GTR+G+I substitution model (automatically determined by the software). The nomenclature used is genotype-subtype-accession number. The graphical representation was generated using the iTOL website.

The classification of HCV is based on the topology of phylogenetic trees derived from viral variant relationships. Until recently, HCV was divided into seven main types based on significant genetic differences (>30% at the nucleotide level) [9,10]. A new type, HCV genotype 8, was recently discovered in India in 2018 [11]. This new type is genetically distinct from the previously known types. Genotypes are further divided into subtypes. Based on the consensus criteria [12], to be confirmed as a new subtype, a virus must have a unique genetic sequence that is different from other known subtypes by at least 15% and sequence information from at least two other isolates in the core/E1 (>90% of the sequence corresponding to positions 869 to 1292 of the H77 reference sequence) and the NS5B region (>90% of positions 8276 to 8615) of the virus’s genome [12]. To date, over 90 confirmed HCV subtypes have been described [10,13]. The virus genotype is indicated by an Arabic number (from 1 to 8), associated with a lower-case letter to indicate the subtype. The phylogenetic tree depicted in Figure 2 illustrates the evolutionary relationships among all known, assigned, provisionally assigned, or unassigned subtypes of the eight HCV genotypes, based on complete genome sequences retrieved from the NCBI Genbank.

### 1.5. HCV Genotypes

Phylogenetic analysis permitted the classification of HCV into 8 major genotypes and 86 subtypes [10]. The global distribution of HCV genotypes and subtypes also varies by geographic region, ethnicity, and risk groups. Genotypes 1 and 3 are the most prevalent, accounting for more than 30% of all infections. Genotypes 2, 4, 5, and 6 account for less than 10% of infections, respectively. Genotype 7 has been found in a few people from Central Africa [9,14].

The HCV genotype distribution varies geographically. Genotype 1 predominates in Europe and North America, followed by genotypes 2 and 3. Genotype 2 is most prevalent in Central Africa, while genotype 3 is common in India, Pakistan, Thailand, other Asian countries, and Northern Europe. Genotypes 4 and 5, likely disseminated through migration and intravenous drug use, are increasing in prevalence. Genotype 4 is primarily found in Central Africa and the Middle East, whereas genotype 5 is more frequently found in Southern Africa. The HCV genotype 6 has a high variation, classified as more than five subtypes. They are highly prevalent in East and Southeast Asia and are mostly found in Laos and Vietnam [5], and northern and northeast Thailand [15].

## 2. HCV Core Antigen (HCVcAg)

### 2.1. Definition and Structure

The HCV core protein, which is released from polyproteins by a signal peptidase (SP), is a 23 kDa precursor protein containing 191 amino acids [16], organized into three distinct regions D1, D2, and D3. This precursor core protein is subsequently processed by a signal peptide peptidase (SPP), producing a mature, two-domain (D1 and D2) protein of approximately 177 amino acids.

**Figure 3:**
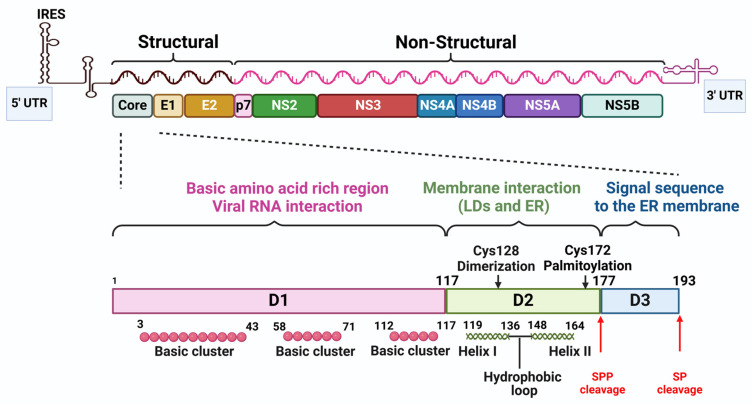
Structure of HCV genome depicting the structural and functional map of an HCV core protein. Created with https://www.biorender.com/ (accessed on 15 September 2024).

The first region, composed of 117 amino acids, is rich in basic residues and contains short hydrophobic segments, binding to the viral RNA. The underlying mechanism is that hydrophobic segments, due to their inherent properties, tend to exclude water molecules. While viral RNA possesses negatively charged phosphate groups, it also contains hydrophobic bases (adenine, guanine, cytosine, and uracil). These hydrophobic bases can engage in favorable interactions with the hydrophobic amino acids present in the core antigen, ultimately resulting in binding. The second region is more hydrophobic and anchors the core protein to lipid droplets (LDs), intracellular structures that store lipids. The final region, containing the signal sequence for another viral protein, is located at the protein’s end. Based on the charge distribution, the D1 domain can be further subdivided into three basic clusters: BD1 (basic domain 1), BD2 (basic domain 2) and BD3 (basic domain 3). The D2 domain contains two amphipathic α-helices, Helix I (HI) and Helix II (HII), separated by a hydrophobic loop (HL) [17] (Figure 3). Mature core proteins typically form clusters at the endoplasmic reticulum (ER) membrane and can assemble into virus-like particles [18]. The D3 region is a short peptide composed of around 15 amino acids, playing a crucial role in signal transduction [19]. Beyond its structural role, the core protein interacts with viral RNA during capsid formation and regulates RNA translation [19]. Additionally, it is implicated in various cellular processes, including apoptosis, lipid metabolism, and liver cancer development. Notably, the core protein can alter the distribution of LDs within cells, which is linked to virus assembly and fat accumulation in the liver. Disrupting the core protein’s interaction with LDs hinders virus production. Furthermore, the core protein influences intracellular calcium levels. Thus, this protein is not only essential for viral structure but also plays a crucial role in regulating cellular functions and contributing to the virus’s life cycle [20].

The current understanding suggests that HCV particles are produced near LDs, where they acquire an envelope in the ER [17] before being released from the cell via the secretory pathway, a process associated with very low-density lipoproteins (VLDLs) production. Research has consistently shown a strong connection between the HCV assembly process and LDs (Figure 3). Even before the development of the HCV cell culture system (HCVcc), studies revealed that the HCV core protein is located on the surface of LDs.

**Figure 4:**
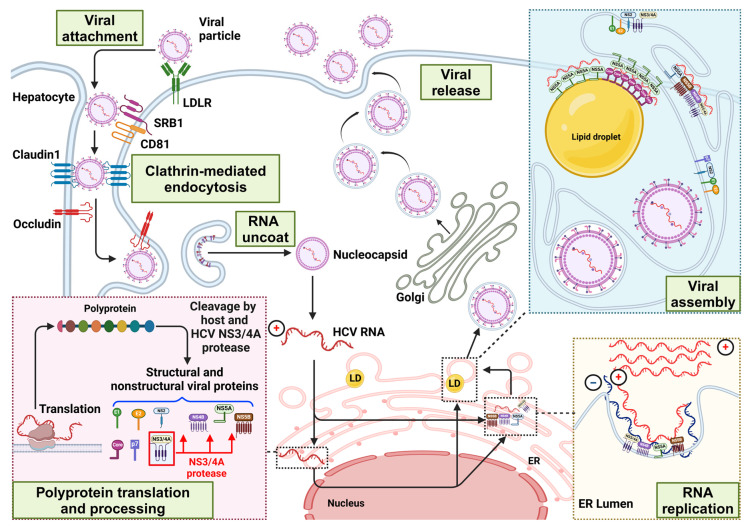
The HCV life cycle. Adapted from [17,21]. Created with https://www.biorender.com/ (accessed on 12 October 2024).

The HCV infection of hepatocytes is a multi-stage process involving interactions with host cell surface proteins, including CD81, SRB1, Claudin 1, and Occludin. Upon entry via endocytosis and fusion with the endosomal membrane, the viral RNA is released into the cytoplasm. Host cell machinery translates this RNA into a polyprotein, from which HCV structural proteins (core, E1, and E2) are liberated from the polyprotein by host proteases. In contrast, the liberation of the six nonstructural proteins (NS2, NS3, NS4A, NS4B, NS5A, and NS5B) relies on a combination of host and viral proteases. These nonstructural proteins possess enzymatic activities essential for the HCV life cycle. Notably, NS2 and NS3 function as viral proteases involved in polyprotein processing, with the NS3/NS4A complex specifically mediating the release of NS5A and NS5B. The replication of the viral RNA genome is primarily mediated by the NS5B polymerase and NS3 helicase. The assembly of progeny virions involves the coordinated interaction of the viral core protein with LDs, facilitating the encapsulation of newly replicated RNA genomes by structural proteins derived from the polyprotein. In addition to its crucial role as a key protein in the replication process, NS5A is believed to be essential for the assembly and release of viral particles. Therefore, NS5A represents a critical target for direct-acting antiviral drugs. Finally, mature virions are released from the hepatocyte, perpetuating the viral life cycle [17,21] (Figure 4).

The viral RNA is packaged into newly formed core oligomers as a result of the interaction between the core and NS5A on or near the LDs. This process is likely to trigger capsid assembly. P7 recruits the core assembly intermediate to the ER membrane, which is essential for the completion of capsid assembly and the initiation of budding. NS2 brings replication and assembly sites into proximity through its interaction with glycoproteins E1 and E2, and NS3/4A (Figure 4). Altering specific regions of the core protein, such as the signal peptide peptidase cleavage site or the D2 domain, impedes the core protein’s localization to LDs and ultimately inhibits the virus’s ability to become infectious [22]. Further investigations using the HCVcc system corroborated that LDs are essential for producing infectious HCV particles and highlighted the crucial roles of the core and NS5A proteins in this process. Both the core and NS5A proteins must be directed to LDs for successful virus production [23].

### 2.2. The Role of HCVcAg in Viral Replication

A major component of the viral particle is the core protein, which interacts with genomic RNA to form the nucleocapsid. After being synthesized on ER membranes and cleaved by the signal peptide peptidase, the core protein forms pairs with itself (homodimerizes) and is then transported to LDs in the cell. The interaction between the HCV core protein and LDs is believed to be crucial for gathering other viral components necessary for building new virus particles. The core protein has a section at its end (C-terminal domain) with two special structures (amphipathic helices) that bind to LDs. As more core proteins accumulate around LDs, the distribution of LDs within the cell changes significantly. Normally, LDs are spread throughout the cell in uninfected cells, but they gather near the cell nucleus when infected with HCV. Importantly, changes to the core protein that prevent it from binding to LDs strongly hinder the virus from forming new particles. Interestingly, when observed live, the core protein quickly moves to LDs and then gradually forms moving clusters (puncta) that travel along microtubules and are likely new virus particles in the process of being released from the cell [17].

It is important to note that the amount of core protein that accumulates on LDs is inversely related to how efficiently the virus produces infectious particles [24]. This might be because the core protein only stays on LDs temporarily before moving to other parts of the cell, where new viruses are assembled. The relationship between the core protein and LDs can also be influenced by proteins from the host cell. For example, an enzyme called diacylglycerol acyltransferase-1 (DGAT1) involved in forming LDs has been shown to interact with the core protein, helping it bind to LDs and allowing the virus to multiply [25]. The movement of the core protein to LDs also requires a cellular enzyme called phospholipase A2 (PLA2G4) that is controlled by another cellular system (MAPK) and its product, arachidonic acid [26]. Surprisingly, another cellular protein called IκB kinase-α (IKK-α) has also been found to be essential for HCV assembly. It has been reported that HCV RNA activates IKK-α, which moves into the cell nucleus and triggers a chain of events involving other cellular proteins (CBP/p300) and regulatory molecules (SREBPs) [27]. This process leads to the increased production of fat-related genes and promotes the formation of LDs with attached core proteins, making it easier for the virus to assemble [17].

The interaction between the core protein and LDs is dynamic, and the core protein needs to be removed from the surface of LDs to move to the place where new viruses bud off. A cellular protein called AP2M1 has been found to interact with a specific sequence (YXXØ motif) on the HCV core protein, which is important for removing the core protein from LDs and leading to virus assembly. However, this finding is difficult to explain because this specific sequence on the core protein is not easily accessible [28,29].

### 2.3. Core Antigen as a Tool for Diagnostic Testing

HCVcAg is a versatile biomarker detectable by a wide array of immunoassay techniques [30,31]. When nucleic acid testing (NAT) for hepatitis C is unavailable due to resource constraints, the WHO recommends using an HCVcAg test to detect active infection [32]. These include traditional methods such as enzyme immunoassay (EIA), chemiluminescence immunoassay (CIA), and radioimmunoassay (RIA), as well as more advanced approaches like recombinant immunoblot assay (RIBA), electrochemical immunosensors (EI), and nanotechnology-based assays [33]. Additionally, rapid point-of-care (POC) testing is enabled through lateral flow assays (LFA), demonstrating the breadth of methodologies available for detecting HCVcAg. In particular, in a study by Wang et al., a highly specific aptamer was used to establish a sensitive method for the detection of the HCV core antigen. The analytical performance of this method was evaluated, revealing impressive detection limits of 10 pg/mL^−1^ when analyzed with a scanner and 100 pg/mL^−1^ when assessed visually [34]. While each method offers distinct advantages, the EIA and CIA techniques are commonly applied by diagnostic companies in their commercial products and are widely used in clinical settings.

**Figure 5:**
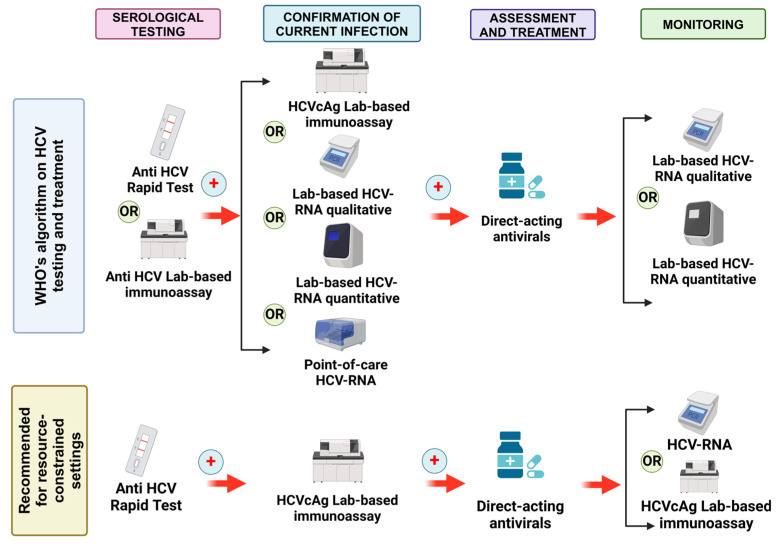
Summary of the algorithm for HCV testing and treatment according to WHO 2022 and recommendations when resources are limited. Adapted from [32,36]. Created with https://www.biorender.com/ (accessed on 15 October 2024).

The WHO’s 2023 updated guidelines on “New recommendation on hepatitis C virus testing and treatment for people at ongoing risk of infection” acknowledge the role of HCVcAg testing [32]. This assay is recognized as one of four options for confirming active HCV infection following a reactive anti-HCV antibody test. According to the 2023 WHO guidelines (Figure 5), the diagnosis of HCV infection before treatment initiation involves a two-step process [32]. The first step, screening, can be performed through rapid diagnostic tests (RDTs) without the need for laboratory infrastructure. However, for confirmatory testing, three options among the four require centralized laboratory assays, including HCVcAg testing, except for POC HCV RNA. It is noteworthy that while the WHO, the Infectious Diseases Society of America (IDSA), and the American Association for the Study of Liver Diseases (AASLD) guidelines do not mention the use of HCVcAg for monitoring sustained virological response (SVR) post-treatment, the European Association for the Study of the Liver (EASL) guidelines acknowledge its role in monitoring after direct-acting antiviral therapy [32,35,36]. For resource-limited settings, a practical approach is recommended: initial screening using serological tests followed by the confirmation of viral infection with HCVcAg. Furthermore, post-treatment monitoring with HCVcAg could also be considered in these settings. The sensitivity, specificity, and correlation of HCVcAg with the gold standard laboratory-based HCV RNA assay will be discussed in detail in subsequent sections.

### 2.4. Comparison of HCVcAg with Other HCV Markers (Anti-HCV, HCV RNA)

Current guidelines from leading global organizations, including the European Association for the Study of the Liver, the Infectious Diseases Society of America, the American Association for the Study of Liver Diseases and the WHO, consistently affirm that HCV RNA testing is the gold standard for diagnosing and monitoring hepatitis C infection [32,35,36,37]. Specifically, the WHO strongly recommends the use of qualitative or quantitative HCV RNA laboratory-based assays. This recommendation stems from the test’s ability to directly detect viral genetic material, thus reflecting viral replication and providing a definitive assessment of an active infection. However, widespread implementation of HCV RNA testing can be challenging due to its technical demands. These include the need for real-time PCR systems, highly trained personnel, and significant financial investment. Consequently, HCVcAg testing, which directly detects viral antigens, has emerged as a recommended alternative for confirming HCV infection. The expression of HCVcAg is highly consistent with that of HCV RNA, but compared with HCV RNA, the detection of HCVcAg is easy to operate, time-saving, and low cost. HCVcAg can be detected within 12~15 days after infection, and the window period can be shortened by 5~7 weeks. In particular, HCVcAg can be detected 1.5 months (38 to 50 days) earlier than anti-HCV and 1 to 2 days after the appearance of HCV RNA in blood samples of newly infected individuals [38] (Table 1). HCVcAg is a serological indicator of virus replication, which can distinguish between previous infection of HCV or current infection. HCVcAg detection is particularly advantageous for immunocompromised individuals, hemodialysis patients, and organ transplant recipients (detail research in Table 2). This stems from the fact that HCV core antigen detection is independent of the host’s immune response, unlike antibody-based assays. HCVcAg can also be used to monitor antiviral efficacy and predict SVR [39]. Table 1 presents a comparative analysis of three serological markers (anti-HCV, HCVcAg, and HCV RNA) recommended within the WHO diagnostic algorithm. This analysis encompasses the key characteristics, advantages, and limitations of each marker.

## 3. Clinical Significance of HCVcAg for Diagnosis and Monitor Treatment

We have reviewed and synthesized data from approximately 80 studies to elucidate the value of the HCVcAg test. Our focus has been primarily on the test’s utility in both HCV diagnosis and treatment monitoring, particularly in the era of direct-acting antiviral agents. While HCV RNA testing remains the gold standard for detecting HCV infection, the HCVcAg test offers a faster and more cost-effective alternative. These findings also collectively support the conclusion that the HCVcAg test provides a reliable and accessible approach for HCV diagnosis, especially in resource-limited settings where RNA-based assays may be less available. The subsequent sections will delve into the significance of HCVcAg testing in diagnostic, treatment monitoring, and predictive applications.

### 3.1. HCVcAg as a Diagnostic Marker for Active Infection

HCVcAg presents multiple advantages for diagnosing active infections. Its earlier appearance compared to HCV antibodies, specific expression during active infection, and ability to differentiate between current and past infections enhance its diagnostic utility. Furthermore, HCVcAg detection remains unaffected by immunosuppression. While exhibiting a strong correlation with HCV RNA levels, HCVcAg assays offer advantages in terms of ease of use, reduced processing time, and lower cost. These characteristics position HCVcAg as a potential alternative to HCV RNA for diagnosing active infection. Combining HCVcAg and antibody serology can improve early detection, accurately stage HCV infection, and monitor the antiviral treatment response, ultimately contributing to improved hepatitis C management.

**Figure 6:**
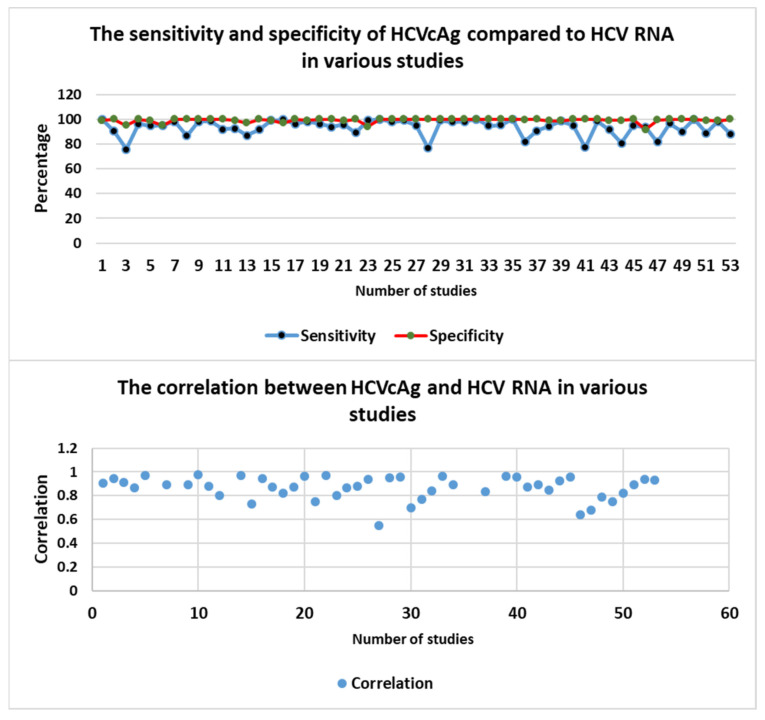
The sensitivity, specificity, and correlation between HCVcAg and HCV RNA in various studies.

Table 2 presents a meta-analysis of 53 studies evaluating the diagnostic performance of HCVcAg testing compared to the gold standard RT-PCR. The HCVcAg assay exhibited excellent sensitivity and specificity. The pooled sensitivity and specificity from studies of HCVcAg were calculated by selecting the overall population sensitivity and specificity in the study and using the Architect HCVcAg assay. The pooled sensitivity was 93.5% (95% CI: 91.8–95.3%), while the pooled specificity was 99.2% (95% CI: 98.7–99.6%). Furthermore, a strong correlation was observed between HCVcAg and HCV RNA levels, with a mean correlation coefficient (*r*) of 0.87 (95% CI: 0.84–0.90). Figure 6 illustrates that approximately 80% of the studies reported a correlation coefficient exceeding 0.8, indicating a strong association between HCVcAg and HCV RNA. Only one study demonstrated a correlation coefficient below 0.6 [40].

Further analysis shows that previous studies predominantly used serum or plasma samples. However, there was no statistically significant difference in the correlation between these two sample types [41,42,43,44,45]. The impact of specific populations on the diagnostic value of HCVcAg assays has also been investigated in several studies. Heidrich et al. investigated the correlation between HCVcAg and HCV RNA in liver and kidney transplant recipients. Their findings demonstrated a strong correlation in both groups, with correlation coefficients of 0.85 and 0.969, respectively [46]. Studies in patients undergoing hemodialysis also showed a high degree of correlation. Wong (Taiwan) and Chuaypen (Thailand) reported correlation coefficients of 0.833 and 0.956, respectively [44,47], in this population. In patients co-infected with HBV and/or HIV, the correlation remained strong compared to the general population with HCV mono-infection [48,49].

On the other hand, this review also examined the use of dried blood spots (DBS) [50,51], which demonstrated a high degree of concordance with serum and plasma samples, although the sensitivity was lower compared to when using the latter two sample types. Furthermore, the impact of storage time on test performance was investigated by Troyano-Hernáez et al. who found that, within a reasonable timeframe, the HCVcAg test quality remained sufficient for diagnostic purposes [52]. However, current guidelines from the EASL do not recommend the use of whole blood or dried blood spots samples for the quantification of HCVcAg in clinical practice [36].

Based on these findings and previous analyses, it can be concluded that HCVcAg testing can effectively replace HCV RNA testing, offering exceptionally high sensitivity and specificity. Moreover, current guidelines (WHO 2023) support this notion, recommending a rapid diagnostic test or laboratory-based immunoassay as the initial step, followed by lab-based HCV RNA (qualitative or quantitative), HCVcAg assays, or POC HCV RNA assays to confirm active infection [32,36].

**Table 2:** A comprehensive review of studies on the value of HCVcAg in diagnosis from 2010 to the present.

No.	Author (Year) [Ref.]	Country (Continent)	Sample Size	HCV Genotype	Medical History	Sample Type	HCVcAg Reagent (LOD cut off)	HCV RNA Viral Load (IU/mL)	Sensitivity (95%CI)	Specificity (95%CI)	Correlation (*p*-Value)
1	Miedouge et al. (2010) [53]	France (Europe)	2913	1, 2, 3, 4, 5, 6	N/A	SR	Architect HCVcAG (3 fmol/L)	0–2000	53.85% (25–81%)	99% (99–100%)	0.9041 (*p* < 0.0001)
								2001–6000	88.24% (64–99%)	99% (99–100%)	
								>6000	100%	99% (99–100%)	
2	Park et al. (2010) [54]	Korea (Asia)	282	1	N/A	SR	Architect HCVcAG (3 fmol/L)	N/A	N/A	N/A	0.9521 (*p* < 0.0001)
				2					N/A	N/A	0.9513 (*p* < 0.0001)
				3					N/A	N/A	0.9429 (*p* = 0.0048)
				Total					90.2% (85–94%)	100% (97–100%)	0.9464 (*p* < 0.0001)
3	Ergunay et al. (2011) [55]	Turkey (Europe)	272	1, 2, 3	N/A	SR	Architect HCVcAG (3 fmol/L)	0–10^3^	N/A	N/A	0.066 (*p* = 0.561)
								10^3^–10^4^	N/A	N/A	0.595 (*p* < 0.001)
								10^4^–10^5^	N/A	N/A	0.404 (*p* = 0.002)
								10^5^–10^6^	N/A	N/A	0.724 (*p* < 0.001)
								≥10^6^	N/A	N/A	0.897 (*p* < 0.001)
								Total	75.8% (66–78%)	95.1% (84–99%)	0.915 (*p* < 0.001)
4	Kesli et al. (2011) [56]	Turkey (Europe)	212	1	N/A	SR	Architect HCVcAG (3 fmol/L)	<10^5^	N/A	N/A	N/A
								≥10^5^	N/A	N/A	0.907 (*p* < 0.0001)
								Total	96.3% (92–99%)	100% (93–100%)	0.864 (*p* < 0.0001)
5	Kuo et al. (2012) [57]	Taiwan (Asia)	405	N/A	N/A	SR	Architect HCVcAG (3 fmol/L)	N/A	97.8 % (93–100%)	97.1% (95–99%)	0.97 (*p* < 0.001)
							Architect HCVcAG (10fmol/L)	N/A	94.6% (90–98%)	98.7% (97–99%)	
6	Alados-Arboledas et al. (2013) [58]	Spain (Europe)	127	N/A	N/A	SR	Architect HCVcAG (3 fmol/L)	N/A	95% (89–99%)	95% (83–99%)	N/A
7	Hadziyannis et al. (2013) [59]	Greece (Europe)	105	1, 2, 3, 4	N/A	SR	Architect HCVcAG (3 fmol/L)	N/A	98% (92–100%)	100% (79–100%)	0.89 (*p* < 0.001)
8	Buket et al. (2014) [60]	Turkey (Europe)	115	N/A	N/A	SR	Architect HCVcAG (3 fmol/L)	N/A	86.5% (78–93%)	100% (82–100%)	N/A
9	Chevaliez (2014) [61]	France (Europe)	188	1	N/A	SR	Architect HCVcAG (3 fmol/L)	N/A	N/A	N/A	0.82 (*p* < 0.0001)
			16	2					N/A	N/A	0.75 (*p* = 0.0009)
			40	3					N/A	N/A	0.92 (*p* < 0.0001)
			61	4					N/A	N/A	0.89 (*p* < 0.0001)
			514	1, 2, 3, 4, 5, 6					98% (96–99%)	100% (98–100%)	0.89 (*p* < 0.0001)
10	Florea et al. (2014) [62]	Romania (Europe)	76	N/A	N/A		Architect HCVcAG (3 fmol/L)	N/A	98.5% (97–99%)	100% (81–100%)	0.98 (*p* < 0.001)
11	Garbuglia et al. (2014) [63]	Italy (Europe)	117	1	100%HIV 3.8%HBV	PM	Architect HCVcAG (3 fmol/L)	N/A	N/A	N/A	0.82
			74	3				N/A	N/A	N/A	0.74
			44	4				N/A	N/A	N/A	0.84
			249	1, 2, 3, 4, others				N/A	92% (78–98%)	100% (80–100%)	0.7420 (*p* = 0.05)
			106	1, 2, 3, 4		PM	Architect HCVcAG (3 fmol/L)	N/A	92% (78–98%)	100 (80–100%)	0.881 (*p* < 0.001)
12	Heidrich et al. (2014) [46]	Germany (Europe)	789	1, 2, 3, others	w/o Tx	SR	Architect HCVcAG (3 fmol/L)	N/A	91% (88–93%)	99% (96–100%)	0.736 (*p* < 0.001)
			185		LTx				98% (94–100%)	100% (90–100%)	0.850 (*p* < 0.001)
			37		NTx				96% (78–100%)	100% (77–100%)	0.969 (*p* < 0.001)
			424		N/A			<10^5^	N/A	N/A	0.001 (*p* = 0.978)
			587					≥10^5^	N/A	N/A	0.805 (*p* < 0.001)
			1011					Total	92.57% (90–94%)	98.94% (97–100%)	0.803 (*p* < 0.001)
13	Kadkhoda et al. (2014) [64]	Canada (America)	154	1, 2, 3, 4	1.3% HIV 0% HBV	SR	Architect HCVcAG (3 fmol/L)	N/A	87% (78–93%)	97% (89–100%)	N/A
14	Reyes-Mendez et al. (2014) [65]	Mexico (America)	211	N/A	N/A	SR	Architect HCVcAG (3 fmol/L)	N/A	91.7% (78–98%)	100% (80–100%)	0.97 (*p* < 0.001)
15	Van Helden et al. (2014) [66]	Germany (Europe)	3558	1, 2, 3	4.4% HIV 6.6% HBV	SR	Architect HCVcAG (3 fmol/L)	N/A	99.0% (98–100%)	99.2% (99–100%)	0.73 (*p* = 0.0003)
16	Kamal et al. (2015) [67]	Egypt (Africa)	410	4	0% HIV 2.2% HBV	SR	Architect HCVcAG (3 fmol/L)	N/A	99.53% (99–100%)	96.81% (93–100%)	0.944 (*p* < 0.001)
17	Demircili et al. (2016) [68]	Turkey (Europe)	189	1, 2, 3, 4	N/A	SR	Architect HCVcAG (3 fmol/L)	N/A	96.2% (89–99%)	100% (97–100%)	0.874 (*p* < 0.01)
18	Medici et al. (2016) [41]	Italy (Europe)	188	N/A	N/A	PM SR	Architect HCVcAG (3 fmol/L)	N/A	98% (92–100%)	99% (94–100%)	0.82 (*p* < 0.001)
18	Alonso et al. (2017) [69]	Spain (Europe)	28	1, 2, 3, 4	35.7% HIV 28.6% HBV	SR	Architect HCVcAG (3 fmol/L)	N/A	96.2% (66–100%)	100% (77–100%)	0.871 (*p* < 0.001)
19	Çetiner et al. (2017) [70]	Turkey (Europe)	26	1, 2, 3, 4	N/A	SR	Architect HCVcAG (3 fmol/L)	0–10^3^	N/A	N/A	−0.294 (*p* = 0.145)
			31					10^3^–10^5^	N/A	N/A	0.815 (*p* < 0.001)
			43					10^5^–10^6^	N/A	N/A	0.785 (*p* < 0.001)
			32					≥10^6^	N/A	N/A	0.755 (*p* < 0.001)
			132					Total	93.75% (88–97%)	100% (83–100%)	0.966 (*p* < 0.001)
20	Duchesne et al. (2017) [48]	Cameroon (Africa)	489	1, 2, 4, others	M-HCV	SR	Architect HCVcAG (3 fmol/L)	N/A	95.7% (94–97%)	98.7% (96–99%)	0.75 (*p* < 0.0001)
			27		HCV-HIV				100% (85–100%)	88.2% (74–96%)	0.84 (*p* < 0.0001)
			28		HCV-HBV				96.4% (79–100%)	98.7% (88–99%)	0.58 (*p* < 0.0001)
			48	1	N/A				97.9% (87–100%)	97.8% (96–99%)	0.60 (*p* < 0.0001)
			41	2					95.1% (82–100%)	97.8% (96–99%)	0.88 (*p* < 0.0001)
			41	4					100% (94–97%)	97.8% (96–99%)	0.75 (*p* < 0.0001)
21	Hullegie et al. (2017) [49]	Netherlands (Europe)	67	1	100% HIV	PM SR	Architect HCVcAG (3 fmol/L)	N/A	89% (75–96%)	100% (85–100%)	0.97 (*p* < 0.001)
22	Mohamed et al. (2017) [71]	Tanzania (Africa)	114	1, 4	43.9% HIV 9.8% HBV	SR	Architect HCVcAG (3 fmol/L)	N/A	99.1% (95–100%)	94.1% (82–99%)	0.80 (*p* < 0.001)
			89			DBS			81.7% (75–87%)	98.9% (94–100%)	0.75 (*p* < 0.001)
23	Rockstroh et al. (2017) [72]	Germany (Europe)	411	1	N/A	PM	Architect HCVcAG (3 fmol/L)	N/A	100% (98–100%)	100% (99–100%)	0.867 (*p* < 0.001)
24	Talal et al. (2017) [73]	USA (America)	109	1, 2, 3, 4	17.4% HIV 100% Opioid use disorder	SR	Architect HCVcAG (3 fmol/L)	N/A	97.9% (89–100%)	100% (94–100%)	0.88 (*p* < 0.01)
25	Wasitthankasem et al. (2017) [74]	Thailand (Asia)	61	1	0.3% HIV 4.5% HBV	PM	Architect HCVcAG (3 fmol/L)	N/A	N/A	N/A	0.905 (*p* < 0.001)
			69	3					N/A	N/A	0.854 (*p* < 0.001)
			90	6					N/A	N/A	0.870 (*p* < 0.001)
			290	1, 3, 6					99% (97–100%)	100% (95–100%)	0.94 (*p* < 0.001)
26	Adland et al. (2018) [40]	UK (Europe)	195	1,3	N/A	N/A	Architect HCVcAG (3 fmol/L)	N/A	95% (89–98%)	100% (95–100%)	0.55 (*p* < 0.0001)
27	Alonso et al. (2018) [75]	Spain (Europe)	204	1, 2, 3, 4	12.3% HIV 27.9% HBV	SR	Architect HCVcAG (3 fmol/L)	N/A	76.6% (95–100%)	100% (95–100%)	0.951 (*p* < 0.001)
28	Benito et al. (2018) [76]	Spain (Europe)	72	N/A	N/A	SR	Architect HCVcAG (3 fmol/L)	N/A	90.91% (80–97%)	100% (80–100%)	N/A
29	Chang et al. (2018) [77]	Taiwan (Asia)	107	1	N/A	SR	Architect HCVcAG (3 fmol/L)	N/A	N/A	N/A	0.945 (*p* < 0.001)
			77	2					N/A	N/A	0.862 (*p* < 0.001)
			221	1,2, others					99.0% (96–100%)	100% (85–100%)	0.960 (*p* < 0.001)
30	Lamoury et al. (2018) [51]	Australia (Australia)	119	1, 2, 3, 6	N/A	PM	Architect HCVcAG (3 fmol/L)	N/A	97.7% (91–100%)	100% (87–100%)	0.695 (*p* < 0.001)
			120			DBS			88.6% (80–94%)	97% (82–100%)	0.649 (*p* < 0.001)
31	Catlett et al. (2019) [50]	Australia (Australia)	186	N/A	N/A	PM	Architect HCVcAG (3 fmol/L)	N/A	98.1% (90–100%) 100% (93–100%)	100% (97–100%)	0.77 (*p* < 0.001)
			186			DBS			90.7% (80–97%) 92.5% (82–98%)	100% (97–100%)	0.82 (*p* < 0.001)
32	Kyuregyan et al. (2019) [78]	Russia (Europe)	31	1, 3, others	N/A	SR	Architect HCVcAG (3 fmol/L)	N/A	100 (79–100%)	100% (78–100%)	0.84 (*p* < 0.005)
33	Pérez-García et al. (2019) [42]	Spain (Europe)	40	1, 2, 3, 4	2.5% HIV	PM SR	Architect HCVcAG (3 fmol/L)	N/A	94.74% (82–99%)	100% (16–100%)	0.965 (*p* < 0.001)
			263						90.62% (83–96%)	98.20% (95–100%)	
34	Suttichaimongkol et al. (2019) [43]	Thailand (Asia)	165	1, 3, 6	1.8% HIV 1.8%HBV	PM SR	Architect HCVcAG (3 fmol/L)	N/A	95.3% (91–98%)	100% (81–100%)	0.8915 (*p* < 0.001)
35	Yu Xiang et al. (2019) [79]	China (Asia)	106	1, 3, 6	N/A	SR	Shandong Laibo Bio-chemical	N/A	55.66% (46–65%)	N/A	N/A
							Architect HCVcAG (3 fmol/L)	N/A	100% (97–100%)	100% (97–100%)	N/A
			46	1a	N/A	SR	Architect HCVcAG (3 fmol/L)	N/A	N/A	N/A	0.894 (*p* = 0.04)
			7	2a					N/A	N/A	−0.360
			18	3a					N/A	N/A	0.400
			3	3b					N/A	N/A	0.500
			9	6a					N/A	N/A	−0.122
			13	1b/3b					N/A	N/A	−0.413
			UD	10					N/A	N/A	0.529
36	Pollock et al. (2020) [80]	UK (Europe)	159	3	5% HIV 0.5%HBV	SR			79% (70–86%)	100% (91–100%)	N/A
			126	1, 2, 4					87% (77–93%)	100% (91–100%)	
			290	1, 2, 3, 4					82.1% (77–86%)	99.8% (99–100%)	
37	Wong et al. (2020) [44]	Malaysia (Asia)	112	N/A	HD patients	PM SR	Architect HCVcAG (3 fmol/L)	N/A	90.7% (83–96%)	100% (87–100%)	0.833 (*p* < 0.001)
38	Abid et al. (2021) [81]	Pakistan (Asia)	200	N/A	N/A	PM	Architect HCVcAG (3 fmol/L)	>12 IU/mL	96.8% (92–99%)	91.8% (83–97%)	N/A
			200					>1000 IU/mL	96.0% (91–99%)	97.2% (90–100%)	
			200					>3000 IU/mL	94.4% (88–98%)	98.6% (92–100%)	
			200				Architect HCVcAG (10 fmol/L)	>12 IU/mL	99.1% (95–100%)	87.6% (78.4–94%)	
			200					>1000 IU/mL	99.1% (95–100%)	93.8% (86–98%)	
			200					>3000 IU/mL	99.1% (95–100%)	97.5% (91–100%)	
39	Chen et al. (2021) [82]	Taiwan (Asia)	412	N/A	N/A	SR	Architect HCVcAG (3 fmol/L)	N/A	98.46% (96–100%)	98.62% (96–100%)	0.964 (*p* < 0.001)
40	Kallala et al. (2021) [45]	Tunisia (Africa)	43	1	N/A	PM SR	Architect HCVcAG (3 fmol/L)	N/A	N/A	N/A	0.966 (*p* < 0.001)
			109	1, 2, 3					95.05% (89–98%)	100% (63–100%)	0.958 (*p* < 0.001)
41	Kannan et al. (2021) [83]	India (Asia)	156	N/A	N/A	SR	Architect HCVcAG (3 fmol/L)	N/A	77.35% (63–87%)	100% (96–100%)	0.872 (*p* < 0.001)
42	Kumar et al. (2021) [84]	Singapore (Asia)	89	1	IH	PM SR	Architect HCVcAG (3 fmol/L)	N/A	98.4% (91–100%)	100% (87–100%)	0.89 (*p* < 0.001)
43	Kumbhar et al. (2021) [85]	India (Asia)	208	N/A	N/A	PM	Architect HCVcAG (3 fmol/L)	N/A	91.58% (84–96%)	99.12% (95–100%)	0.85 (*p* < 0.0001)
44	Ponnuvel et al. (2021) [86]	India (Asia)	140	1, 3, 4	1.42% HIV 5.7%HBV	PM	Architect HCVcAG (3 fmol/L)	N/A	80.62% (74–88%)	99% (95–100%)	0.927 (*p* < 0.0001)
45	Chuaypen et al. (2022) [47]	Thailand (Asia)	93	1, 2, 3, 6	HD patient 0%HIV 0%HBV	SR	Architect HCVcAG (3 fmol/L)	N/A	94.9% (86–99%)	100% (90–100%)	0.956 (*p* < 0.001)
46	Le et al. (2022) [87]	Vietnam (Asia)	102	N/A	N/A	SR	Architect HCVcAG (3 fmol/L)	N/A	93.33% (78–99%)	91.67% (83–97%)	0.64 (*p* < 0.001)
47	Sun et al. (2022) [88]	Taiwan (Asia)	24	1	100% HIV 9.9%HBV	PM	Architect HCVcAG (3 fmol/L)	N/A	87.1% (77–94%)	99.4% (99–100%)	0.355 (*p* = 0.089)
			13	2							0.806 (*p* = 0.001)
			22	6							0.875 (*p* < 0.001)
48	Ponnuvel et al. (2023) [89]	India (Asia)	383	1, 2, 4, 6	N/A	PM	Architect HCVcAG (3 fmol/L)	N/A	96.61% (94–98%)	100% (99–100%)	0.79 (*p* < 0.0001)
			40	1, 2, 4	N/A	PM			92.50% (80–98%)	100% (91–100%)	0.88 (*p* < 0.0001)
49	Eid et al. (2023) [90]	Egypt (Africa)	50	N/A	N/A	PM	ELISA MyBioSource (3 fmol/L)	N/A	90% (76–97%)	100% (69–100%)	0.753 (*p* < 0.001)
50	Troyano-Hernáez et al. (2023) [52]	Spain (Europe)	70	1, 2, 3, 4, others	N/A	SR	Architect HCVcAG (3 fmol/L)	POC PCR Xpert HCV VL	100% (93–100%)	100% (83–100%)	0.82 (*p* < 0.001)
						DBS			100% (93–100%)	100% (83–100%)	0.90 (*p* < 0.001)
									N/A	N/A	0.90 (*p* < 0.001)
									N/A	N/A	0.82 (*p* < 0.001)
									N/A	N/A	0.81 (*p* < 0.001)
51	Vieira et al. (2023) [91]	Portugal (Europe)	131	N/A	N/A	SR	Architect HCVcAG (3 fmol/L)	<1000 IU/mL	50%	99.1%	N/A
								≥1000 IU/mL	93.8%	-	N/A
								Total	88.9% (74–97%)	99.1% (95–100%)	0.89 (*p* < 0.001)
52	Naveed et al. (2024) [92]	Pakistan (Asia)	394	3	0% HIV 0.2%HBV	PM	Architect HCVcAG (3 fmol/L)	N/A	98.0% (95–99%)	98.6% (95–100%)	0.935 (*p* < 0.001)
53	Garg et al. (2024) [93]	India (Asia)	90	N/A	N/A	PM	Architect HCVcAG (3 fmol/L)	N/A	88.33% (77–95%)	100.00% (88–100%)	0.93 (*p* < 0.0001)

Abbreviation: CI: confidence interval; HBV: hepatitis B virus; HCV: hepatitis C virus; HIV: human immunodeficiency virus; HD: hemodialysis; IH: immunocompromised hosts; LTx: liver transplantation; NTx: kidney transplantation; N/A: no data available; POC: point of care; PM: plasma, SR: serum; Ref.: reference; UK: United Kingdom; USA: United States of America; w/o Tx: without transplantation.

### 3.2. HCVcAg as a Tool for Monitoring Treatment Response

In evaluating the monitoring value of HCVcAg versus HCV RNA during treatment, studies commonly report correlation coefficients, kappa indices, or concordance rates. These studies consistently demonstrate that HCVcAg levels decline more rapidly than HCV RNA levels following the initiation of treatment [94]. However, the timing of assessments and the specific parameters monitored can vary across studies. Notably, a strong correlation between HCVcAg and the gold standard is often observed at the baseline [95,96,97,98]. During treatment, this correlation becomes more variable, possibly due to the rapid decline in viral load and differential responses based on genotype and host factors [94,95,99]. Nevertheless, after treatment completion, the correlation strengthens, particularly at the 12- and 24-week sustained virologic response (SVR12 and SVR24) time points. In terms of agreement, the concordance between HCVcAg and the gold standard at the baseline and post-treatment is generally high, as indicated by correlation coefficients of 0.7 or greater, as shown in Table 3. Additionally, kappa indices and concordance rates further support the utility of HCVcAg in monitoring treatment, especially in resource-limited settings. While the 2022 WHO guidelines do not specifically mention using HCVcAg to monitor DAA treatment, the EASL recommends that an undetectable HCV core antigen level at 12 or 24 weeks post-treatment can serve as a substitute for HCV RNA testing to determine SVR12 and SVR24, respectively. This recommendation applies to patients who had detectable HCV core antigen levels before starting treatment. The widespread adoption and implementation of this assay for monitoring treatment response is crucial for achieving the goal of HCV elimination by 2030, particularly in resource-limited settings.

### 3.3. The Advantages of HCVcAg Assays in Countries with Low-Resource Settings

HCVcAg assays offer several advantages in resource-limited settings, making them valuable tools in the fight against hepatitis C and achieving HCV elimination by 2030. First, the performance of the assay was noteworthy. As summarized in Table 2, most studies demonstrated a good to excellent correlation between the HCVcAg and the gold standard HCV RNA assay. Second, HCVcAg assays are generally more cost-effective than NAT, the gold standard for HCV diagnosis. This makes them more accessible in settings with limited healthcare budgets. Specifically, Reyes-Mendez et al. demonstrated the economic benefits of using HCVcAg as a confirmatory test compared to HCV RNA. However, this benefit was more pronounced (USD 6.98 vs. USD 10.31) in areas with high HCV prevalence. In low prevalence settings, the cost advantage was less significant (USD 6.37 vs. USD 8.63) [65]. Additionally, Kamal et al. reported that using HCVcAg to monitor treatments throughout their course resulted in cost savings of USD 860 compared to HCV RNA PCR monitoring. A combined HCVcAg and HCV RNA monitoring strategy also yielded USD 493.8 in savings compared with PCR alone [67]. Wasitthankasem et al. compared the costs of two testing strategies. The first strategy involved immediate HCV RNA testing following a positive anti-HCV antibody test, with a total cost of USD 19,655. The second strategy involved confirming active infection with HCVcAg in samples with an anti-HCV antibody signal-to-cutoff ratio (S/CO) ≥ 5.0. Only samples with reactive anti-HCV antibodies and non-reactive HCVcAg were confirmed by HCV RNA testing; the total cost for this strategy was USD 10,213 [74]. The economic value of HCVcAg is further highlighted by its simplified workflow and facilities compared to NAT, reducing the need for specialized equipment and highly trained personnel. This lowered the overall testing cost. Third, the accessibility of HCVcAg is expanding, with DBS testing simplifying sample collection, storage, and transport, which is crucial in areas with limited infrastructure. Troyano-Hernáez et al. demonstrated a good correlation between HCVcAg and HCV RNA in dried serum and DBS samples [52], although these sample types are not yet widely recommended for use. The reasons for this could be a lower analyte concentration in the DBS sample, variability introduced during collection and processing, potential effects on analyte stability during drying and storage, matrix effects from blood components, and less efficient elution of the antigen from the filter paper. These factors can collectively contribute to a reduced ability to accurately measure HCVcAg in DBS compared to plasma.

### 3.4. Weaknesses of Using HCVcAg in the Test-to-Treat Approach for HCV Infection

Similar to HCV RNA testing, HCVcAg can be used to detect active infections to initiate treatment. Although HCVcAg has a slightly lower sensitivity, in practice, its use in the field is not much different from NAT or RNA detection. The HCVcAg test is an immunoassay that requires specific equipments from the manufacturer to achieve rapid and accurate results. It cannot be performed manually at the point of care, and when performed in small quantities, testing costs increase because of the need for positive and negative controls for each run. Furthermore, the equipment or machines are typically available only in large hospitals. Currently, the market is a monopoly, with limited companies providing this test, and it is not suitable for POC testing. In contrast, there are qualitative HCV RNA tests at POC options, which makes them easier to use than HCVcAg. These POC devices utilize reverse transcriptase polymerase chain reaction technology to quickly identify the presence of the hepatitis C virus. With just a small blood sample from a fingertip, these tests can detect HCV RNA and provide results in approximately an hour. This rapid and convenient diagnostic capability is crucial for expanding access to HCV testing and facilitating timely treatment interventions, especially in decentralized healthcare settings.

In Thailand, the National Health Security Office (NHSO) issued guidelines in 2023 allowing the use of qualitative or quantitative HCV RNA tests as well as HCVcAg tests for diagnosis, treatment, and monitoring of treatment outcomes. In clinical practice, most healthcare providers rely on HCV RNA testing to initiate and monitor treatment. Despite advancements in combined HCVcAg and anti-HCV testing (Duo test), which is being developed for blood donor screening and to reduce the window period, the sensitivity of HCVcAg in the Duo test remains suboptimal. Specifically, a study by Ananchuensook et al. demonstrated that the sensitivity of the HCVcAg assay within the Duo test for diagnosing active infection and monitoring sustained virologic response (SVR) was 87.50% and 57.14%, respectively [106]. Similarly, Kanokudom et al. evaluated the Duo test on 769 samples with various genotypes (1,3,6) and reported a sensitivity of 70.6% and a specificity of 100% for Duo/HCVcAg. Furthermore, this study found no statistically significant correlation between Duo/HCVcAg and HCV RNA [107]. These findings suggest that the Duo test may have value in the diagnosis of chronic HCV infection. However, to enhance its utility in identifying active infection, further development is needed to improve the sensitivity of both the Duo test and standalone HCVcAg assays. Enhanced sensitivity, particularly in correlation with HCV RNA levels, will facilitate better-informed clinical decision-making and improve access to timely treatment. Therefore, despite the aforementioned strengths and limitations of HCVcAg in terms of sensitivity, leading global organizations (EASL, IDSA, AASLD, and the WHO) still consider it an alternative tool, with HCV RNA testing remaining the gold standard in clinical practice.

### 3.5. Challenges and Limitations of HCVcAg Testing

Although HCVcAg assays offer numerous advantages, they also present limitations and challenges that may hinder their widespread adoption in clinical practice. Firstly, the sensitivity of the assay is significantly influenced by viral load. This is evident in Table 3, which shows a weak correlation between HCVcAg and HCV RNA during treatment and post-treatment follow-up with DAAs. Although studies have reported a high correlation in patients achieving SVR, the latest WHO guidelines (2022) recommend qualitative or quantitative HCV RNA testing for SVR monitoring. Secondly, assay specificity can be affected by the HCV genotype and subtype diversity. Sun et al. reported a lower correlation for genotype 1 compared to genotypes 2 and 3, possibly due to the quasispecies nature of HCV, suggesting that further investigation is needed [88]. Data from Pollock et al. revealed that a significant proportion of HCV-infected individuals may be missed, with genotype 3 associated with an increased rate of false-negative results (OR = 3.59, 95% CI: 1.32–9.71). These findings have implications for HCV diagnosis and treatment response monitoring, particularly in low- and middle-income regions where genotype 3 is prevalent [80]. Furthermore, the high genetic variability of HCV can lead to amino acid changes in viral proteins, such as the core protein, potentially affecting their recognition by commercially available immunoassays. In a comprehensive study by Hansoongnern et al. (2023) [108], a strong positive correlation between HCV RNA and HCVcAg was observed across all HCV genotypes (1a, 1b, 3a, and 6), with correlation coefficients ranging from 0.88 to 0.96 (*p* < 0.001). However, some samples, particularly those with genotypes 3a and 6, exhibited lower-than-expected HCVcAg levels compared to their corresponding HCV RNA values. Sequence analysis revealed that these samples carried amino acid substitutions at position 49 of the core protein, replacing threonine with alanine or valine. Mutations at this site may interfere with HCVcAg recognition by anti-HCV monoclonal antibodies [108]. Thirdly, the cost-effectiveness of HCVcAg testing is most pronounced in populations with high HCV prevalence. Reyes et al. demonstrated that the cost of HCVcAg testing was USD 6.98 compared to USD 10.31 for HCV RNA in high-prevalence settings. In low prevalence areas, the cost advantage was less substantial (USD 6.37 vs. USD 8.63) or even negligible [65]. Finally, WHO recommends laboratory-based HCVcAg assays for confirmatory diagnosis, which are typically performed on large platforms and may be difficult to implement in POC settings. In addition, immunoassays require calibration and internal quality control procedures. Operating small batches of tests while maintaining continuous quality assurance can increase the cost per test.

## 4. The Prospects in the Clinical Application of HCVcAg

### 4.1. Predicting Disease Progression and Liver Damage

HCV infection can promote hepatic fibrosis through a complex molecular mechanism involving the upregulation of both transforming growth factor beta 1 (TGF-β1) and microRNA-192 (miR-192). This process is mediated by the HCV core protein, which stimulates the expression of miR-192 [109]. Following upregulation, miR-192 is packaged into exosomes and transported to hematopoietic stem cells (HSCs) in the liver. The delivery of miR-192 to HSCs triggers the activation and subsequent production of fibrogenic markers, contributing to the development of liver fibrosis [110].

The HCV core protein, an RNA-binding protein, exhibits multiple functions that contribute to HCV pathogenesis. It can independently induce hepatic steatosis, insulin resistance, and HCC [111]. Furthermore, it stimulates hepatocyte proliferation and specific mutations within the core protein in genotype 1 patients associated with an elevated risk of HCC even after viral eradication [112]. The HCV NS3 protein promotes hepatic fibrosis in chronic liver disease [113], whereas NS5A plays a central role in viral replication and assembly. NS5 also contributes to hepatic steatosis, inhibits apoptosis, and confers resistance to interferon-α therapy. The progression of chronic HCV infection to HCC involves key cellular and molecular pathways, including the epidermal growth factor pathway [114], signal transducer and activator of the transcription 3 pathway, transforming growth factor beta pathway, and vascular endothelial growth factor (VEGF) pathway [115].

The HCV core protein exhibits multifaceted functionality, notably its ability to interact with cellular proto-oncogenes and disrupt their normal expression patterns. This disruption contributes significantly to the development of HCC, a common and serious consequence of chronic HCV infection. Extensive research has highlighted the core protein’s pivotal role in manipulating various signaling pathways crucial to HCC development. These pathways include transforming growth factor β (TGF-β), nuclear factor κB (NF-κB), tumor necrosis factor α (TNF-α), cyclooxygenase-2 (COX-2), Wnt/β-catenin (WNT), vascular endothelial growth factor (VEGF), and peroxisome proliferator-activated receptor α (PPARα) [116,117,118]. The intricate mechanisms by which the core protein modulates these pathways remain an area of active investigation. A deeper understanding of the molecular interplay between the HCV core protein and these signaling pathways is essential for developing effective strategies to prevent HCV-related HCC. Given its involvement in HCC pathogenesis, the HCV core antigen (HCVcAg) holds promise as a valuable molecular target for both research and clinical monitoring. By tracking HCVcAg levels, clinicians can potentially identify individuals at a higher risk of developing adverse hepatic outcomes, such as cirrhosis and HCC, following HCV infection. This knowledge could facilitate early intervention and improve patient outcomes.

### 4.2. Simplifying HCV Diagnosis

While HCVcAg is recommended as a biomarker for both diagnosis and treatment monitoring in reputable guidelines, its use remains limited. Current HCVcAg assays require centralized laboratory testing with immunoassay systems and are restricted to serum or plasma samples. To achieve HCV elimination by 2030, a key strategy involves simplifying the diagnostic process to facilitate earlier treatment initiation with DAAs.

The WHO has proposed a combined anti-HCV and HCVcAg assay to optimize the diagnostic pathway and enhance accuracy [119]. Furthermore, the development of RDTs or POC tests represents a critical trend and strategy for expanding HCVcAg testing. Addressing these needs, Daktari Diagnostics, based in Boston, is developing a novel, rapid, and affordable POC diagnostic tool for HCV. This system utilizes microfluidic technology to detect HCVcAg from a single drop of blood, delivering results within 30 min using a portable cartridge [120]. This technology, also under development for HIV, holds significant promise for resource-constrained settings, particularly in low- and middle-income countries. Despite its promising potential, HCV presents a challenge due to its highly diverse nature. Continuous mutations within the viral genome can alter the structure of the core protein, potentially hindering its detection by antibodies in diagnostic kits, leading to false-negative results. As demonstrated by Hansoongnern et al., amino acid changes in the core protein were observed in HCVcAg-negative samples [108]. Encouragingly, according to author Chen, a strong correlation persists between HCVcAg and HCV RNA, even in the presence of common resistance-associated substitutions (RASs) like Y93H, V179I, Q80K, S122G, and C316N, which were prevalent in the era of DAAs [121]. This suggests that developing highly accurate HCVcAg assays that can adapt to these viral variations remains a crucial strategy for achieving HCV elimination.

## 5. Conclusions and Potential Future Applications

HCVcAg has significant potential for expanding access to hepatitis C diagnosis and care, especially in resource-limited settings. As a valuable tool for screening, diagnosing, and monitoring hepatitis C, HCVcAg closely aligns with HCV RNA levels, offering a cost-effective and efficient alternative for managing the infection. While its sensitivity can be influenced by genetic variations in the core protein across different genotypes, ongoing advancements in test sensitivity, standardization, and POC applications make HCVcAg testing increasingly vital. This is particularly true for low- and middle-income countries striving to meet the WHO’s 2030 hepatitis C elimination goals. Beyond its role in diagnosis and monitoring, HCVcAg also impacts liver disease by contributing to liver fibrosis and affecting pathways that can lead to liver cancer.

Looking ahead, with the application of advancements in science and technology, particularly artificial intelligence, HCVcAg is expected to play an even greater role in treatment monitoring and potentially even in predicting treatment response. An example lies in the advancements in nanotechnology, which offers several key advantages over traditional methods like EIA. Firstly, they typically exhibit a higher sensitivity, enabling the detection of lower concentrations of target molecules, such as viral antigens or antibodies. This is attributed to the unique properties of nanomaterials, allowing for signal amplification and improved binding efficiency. Secondly, nanotechnology-based assays can be faster and require smaller sample volumes, leading to quicker turnaround times and reduced costs. Thirdly, these assays often demonstrate enhanced specificity, minimizing cross-reactivity and false-positive results. Finally, nanotechnology allows for the development of flexible and miniaturized platforms, facilitating point-of-care testing and integration with lab-on-a-chip devices. Together, these advantages position nanotechnology-based assays as powerful tools for disease diagnosis and monitoring. In conclusion, as technology and artificial intelligence progress, leading to decreased costs and wider accessibility, HCVcAg testing is poised to become a cornerstone in global efforts to eliminate hepatitis C.

## Figures and Tables

**Table 1:** Comparison of Anti-HCV, HCVcAg, and HCV RNA Tests.

Characteristic	Anti-HCV	HCVcAg	HCV RNA
Nature	Protein-Antibody	Protein-Antigen	Nucleic acid
Origin	HCV-infected individual	Virus	Virus
Time of appearance in blood after infection	8–11 weeks after exposure	Within 12–15 days after exposure	Approximately 2 weeks after exposure
Persistence in Blood	Virtually lifelong	Disappears after successful treatment or clearance of HCV (usually within 6 months)	Disappears after successful treatment or clearance of HCV (usually within 6 months)
Testing principle	Point-of-care: Lateral flow immunoassay Laboratory-based systems: Enzyme Immunoassay or Chemiluminescence Immunoassay Fluorescence immunoassays	Point-of-care: Lateral flow immunoassay (Not commercially available) Laboratory-based systems: Enzyme Immunoassay or Chemiluminescence Immunoassay	Point-of-care: Reverse transcription polymerase chain reaction. Laboratory-based systems: Reverse transcription polymerase chain reaction
Advantages	HCV exposure marker: Indicates past or current HCV contact. Persistent positivity: Remains positive even after successful HCV clearance. Accessible testing: Cost-effective, simple to administer, and easily implemented on a large scale.	Cost-effective: Lower cost than HCV RNA testing. Simplified testing: Easier to perform and less labor-intensive. Flexible sample handling: No need for immediate testing after sample collection; can be detected in dried blood spots. Comparable information: Provides similar information to HCV RNA testing regarding active infection. Good correlation with the gold standard (HCV RNA).	Remains the gold standard for diagnosis and treatment monitoring according to guidelines from reputable associations and organizations. Both qualitative, quantitative, and rapid tests are accepted according to WHO guidelines. Can quantify viral load
Limitations	No infection status differentiation: Does not distinguish between active infection and resolved infection. Low sensitivity in early infection (window period). Cannot quantify viral load.	Less sensitive than HCV RNA, chances of missed detection. May have false positives or false negatives in certain conditions. Laboratory-based tests require expensive automated systems. Rapid tests are not yet approved.	High cost per test. Requires advanced equipment systems. Requires highly skilled technical personnel. Difficult to implement widely in resource-limited areas.

**Table 3:** A comprehensive review of studies on the value of HCVcAg in treatment monitoring from 2016 to the present.

No.	Author (Year) [Ref.]	Country (Continent)	Sample Size	HCV Genotype	Co-Infection	Sample Type	HCVcAg Reagent	Treatment Point	Kappa/ Concordant	Correlation (*p*-Value)
1	Nguyen et al. (2016) [95]	Ireland (Europe)	110	1	N/A	PM SR	Architect HCVcAG	Baseline	N/A	0.882 (*p* < 0.001)
								Week 1	N/A	0.743
								Week 2	N/A	0.678
								Week 4	N/A	0.505
			42	1	N/A	PM SR		Baseline	N/A	0.785
								Week 4	N/A	0.952
2	Alonso et al. (2017) [69]	Spain (Europe)	28	1, 2, 3, 4	35.7% HIV 28.6% HIV	SR	Architect HCVcAG	EOT	0.96	0.871
3	Arboledas et al. (2017) [99]	Spain (Europe)	262	1, 2, 3, 4	31.3% HIV	PM	Architect HCVcAG	Baseline	0.396	N/A
								Week 1	0.175	N/A
								Week 4	0.225	N/A
								EOT	0.386	N/A
								PT	1.0	N/A
4	Loggi et al. (2017) [96]	Italy (Europe)	96	1, 2, 3, 4	36% HBV	SR	Architect HCVcAG	Baseline	N/A	0.767 (*p* < 0.0001)
								Overall	0.62	N/A
5	Rockstroh et al. (2017) [72]	Germany (Europe)	411	1	N/A	PM	Architect HCVcAG	Baseline	99.5%	0.867 (*p* < 0.0001)
								PT Week 4	96.46%	
								PT Week 12	99.75%	
6		France (Europe)	631	1	N/A	PM	Architect HCVcAG	Overall	N/A	0.867 (*p* < 0.0001)
7	Chevaliez et al. (2018) [100] van Tilborg et al. (2018) [101]	Canada, Germany, and the USA (America and Europe)	219	1, 2, 3, 4, 5,6	1.4% HBV	SR	Architect HCVcAG	Baseline	N/A	0.72 (*p* < 0.0001)
								Week 4	N/A	0.16 (*p* = 0.04)
								EOT	N/A	0.93 (*p* < 0.0001)
								PT Week 12	N/A	0.97 (*p* < 0.0001)
								PT Week 24	N/A	0.97 (*p* < 0.0001)
8	Łucejko et al. (2019) [102]	Poland (Europe)	514	N/A	N/A	PM	Architect HCVcAG	Baseline	98.1%,	0.75 (*p* < 0.0001)
								EOT	98.9%	N/A
								PT Week 12	98.7%	N/A
9		Taiwan (Asia)	110	1	7%HBV	SR	Architect HCVcAG	Baseline	96.5%	0.879 (*p* < 0.001)
								Week 2	36.6%	N/A
								Week 4	47.7%	N/A
								EOT	84.1%	N/A
								PT Week 12	100%	N/A
10	Maneerat Chayanupatkul et al. (2020) [103]	Thailand (Asia)	101	1	36.6% HIV	SR	Architect HCVcAG	Baseline	N/A	0.867 (*p* < 0.001)
								SVR 12	N/A	0.996 (*p* < 0.001)
								SVR 24	N/A	0.994 (*p* < 0.001)
11	Nouh et al. (2020) [104]	Egypt (Africa)	48	N/A	N/A	SR	Quick Titer™ (Glory Science) HCVcAG	Baseline	N/A	0.677 (*p* < 0.001)
12	Mancebo et al. (2021) [98]	Spain (Europe)	274	1, 2, 3, 4, 5	N/A	PM	Architect HCVcAG	Baseline	N/A	0.832 (*p* < 0.01)
								PT Week 12	N/A	0.775 (*p* < 0.01)
13	Rossetti et al. (2021) [97]	Italy (Europe)	180	1, 2, 3, 4	N/A	PM SR	Architect HCVcAG	Baseline	0.52	0.8 (*p* < 0.01)
								Overall		0.75 (*p* < 0.01)
14	Ko et al. (2022) [105]	Taiwan (Asia)	98	1, 2, 6	N/A	PM	Architect HCVcAG	Baseline	N/A	0.951 (*p* < 0.001)
								PT Week 12	N/A	
								PT Week 48	N/A	
15	Vieira et al. (2023) [91]	Portugal (Europe)	131	N/A	N/A	SR	Architect HCVcAG	Treatment	0.789	N/A
								EOT	0.638	N/A
								SVR 12	1.000	N/A
16	Jaya Garg et al. (2024) [93]	India (Asia)	90	N/A	N/A	PM	Architect HCVcAG	Baseline	N/A	0.93 (*p* < 0.0001)
								PT Week 2	N/A	0.94 (*p* < 0.0001)
								PT Week 4	N/A	0.91 (*p* < 0.0001)
								PT Week 8	N/A	0.82 (*p* < 0.0001)
								PT Week 12	N/A	0.82 (*p* < 0.0001)

Abbreviations: EOT: end of treatment; HBV: hepatitis B virus; HCV: hepatitis C virus; HIV: human immunodeficiency virus; N/A: no data available; PM: plasma, SR: serum; USA: United States of America; Ref.: reference; PT: post-treatment; SVR: sustained virological response.

## Data Availability

Not applicable.
